# Body and Social Anhedonia of Depression: A Bifactor Model Analysis

**DOI:** 10.5334/pb.524

**Published:** 2020-04-02

**Authors:** Bo Feng, Yuan Jiang, Yijun Li, Xufeng Liu, Shengjun Wu

**Affiliations:** 1 Department of Military Medical Psychology, Air Force Medical University, Xi’an, CN

**Keywords:** depression, anhedonia, bifactor model analysis

## Abstract

The purpose of this study was to develop a statistical model of anhedonia of depression. The study included 748 healthy controls (350 women, 46.79%, age 21.27 ± 4.72) and 80 patients (32 women, 40%, age 38.36 ± 16.42). The Physical Anhedonia Scale (PhAS), the Social Anhedonia Scale (CSAS), the Positive and Negative Affect Scale (PANAS) and the Beck Depression Inventory(BDI) were administered. Classical Measurement Theory (CTT) and Item Response Theory (IRT) were applied to the collected data. We observed that the general factor of depression status was significantly related with positive emotion (r = –0.37, P < 0.05), negative emotion (r = 0.62, P < 0.05) and BDI (r = 0.48, P < 0.01). A significant difference also was observed between controls and patients. The bifactor model of anhedonia of depression provided a better fit to the data than a unidimensional model. The bifactor model appears to be useful to describe anhedonia in depression.

## Introduction

Depression is a high-prevalent and debilitating mental disorder associated with low mood, anhedonia, and alterations in behavior and emotional processing (Kaiser, Andrews-Hanna, Wager & Pizzagalli, 2015). The global point prevalence and lifetime prevalence of depressive symptoms are 12.9% and 10.8% respectively (Lim, Tam & Lu, 2018). Anhedonia and depressed mood are core diagnostic criteria for a major depressive episode (MDE) which is a part of MDD according to the Diagnostic and Statistical Manual of Mental Disorders (Fifth Edition, DSM-5) (Marty & Segal, 2015). According to Pelizza’s study, there were one-third depression patients experienced apparently anhedonia (Pelizza & Ferrari, 2009). Anhedonia refers to the reduction of the ability to experience pleasure (Loas, 1996), and there is emotional function damage to anhedonia individuals. The subjective experience of positive emotional stimulation is reduced (positive emotion is weakened), and emotional impairment will further affect social function. In Bethany’s study, they found that lower self-reported position was associated with poor course of MDD (Morris, Bylsma & Rottenberg, 2009). The DSM-5 defines anhedonia as the “diminished interest or pleasure in nearly all activities” which often clinically presented as decreased motivational drive and consummatory pleasure (Barbano & Cador, 2006). Previous research had indicated that approximately 70% of individuals with MDD experience clinically significant anhedonic symptoms, making it more challenging for patients to achieve functional recovery (Shankman et al., 2010). What’s more, anhedonia predicts poorer drug treatment response among both adults and adolescents (Uher et al., 2012; Mcmakin et al., 2012).

Different categories of anhedonia can be measured by some outstanding questionnaires such as FCPCS, SHAPS and TEPS (Fawcett, Clark et al., 1983; Snaith et al., 1995; Gard et al., 2006), what’s more, the Chapman physcial anhedonia scale (PhAS) and the Chapman social anhedonia scale (SAS) were the most widely used questionnaires (Kosmadakis et al., 1995; Chapman et al., 1976; Horan et al., 2008). In the past, most of the studies independently discussed physical anhedonia (PhA) or social anhedonia (SA) composition, but lacked in-depth analysis of its internal structure. There is no doubt that PhA and SA are two unique components of pleasure impairment (Chapman et al., 1976), but as two major trait dimensions, the Chapman scale essentially measure a kind of pleasure ability. After reviewing the literature, it’s found that there was a high correlation between PhA and SA (Horan et al., 2008), which comprehensively reflected the all-sided emotion management defect. The ability to experience physical pleasure and social pleasure should not be superfluous to each other (Cohen et al., 2011). These two components were usually combined to perform their functions in mental illness. Due to the initial limitations of the scale, the researchers had to select the “true anhedonia” subgroup of subjects who had reached the cut-off point of PhA and SA scores simultaneously (Pelizza & Ferrari, 2009), while PhA and SA scores were generally positively correlated. There was still a debate about whether the two components reflect consistent psychological attributes (Berenbaum & Oltmanns, 1992). The core role of anhedonia may be derived from the impairment of the ability to experience pleasure itself, rather than PhA or SA. These two dimensions should have internal links and related attributes that require in-depth structural analysis. The first purpose of our research was to verify the measurement validity of Chapman anhedonia questionnaire in Chinese clinical and non-clinical population. More importantly, we used bifactor analysis to extract the common components of the two, which was of great value for further understanding the nature of psychopathological trait emotion.

Item Response Theory (IRT) is a theory that uses mathematical modeling to improve the accuracy of psychological measurement. IRT has been widely used in various fields of psychological measurement, including intelligence tests and clinical symptom assessment. Especially, the bifactor model which was newly developed had the performance in improving clinical psychological symptom assessment deserves more attention (Gibbons & Hedeker, 1992). Bifactor model not only improved the accuracy of measurement tools, but also enabled us to have a deeper understanding of the characteristics of psychological symptoms (Thomas, 2011). Bifactor model is a hierarchical model which is assumed that the symptoms of several related psychological disorders are determined by two factors, one is general factor and the other is specific factor. Simms indicated that there was consensus that the large role of general distress or negative affect (NA) plays in the mood and anxiety disorders (Simms et al., 2008). What’s more, they recognized that general factors were insufficient to explain the observed heterogeneity among individuals with emotional or anxiety problems. Instead, most models contain the acknowledgement that specific components may exist to help distinguish between symptom groups (e.g. panic and generalized anxiety) that have universal pain or NA as a component. Finally, these models typically include a hierarchical structure characterized by a general factor at the top (e.g., general distress, NA, internalizing) that subsumes multiple specific symptom facets at the lower level (Simms, Daniel, Watson & O’Hara, 2008).

For anhedonia, PhA and SA are different clinical manifestations. They should be determined by general anhedonia factors and reflect specific factors of physical anhedonia and social anhedonia to some extent. Elucidating this relationship would be helpful in understanding the nature of anhedonia and its role in psychiatric disorders such as depression. Only through this precise analysis, accurate measurement and effective intervention can be achieved. In order to tease out anhedonia and its clinical manifestations, we used hierarchical bifactor model to extract the general anhedonia components from the PhA and SA. In addition, this research compared the statistical relationship between PhA and SA and clinical indicators (depression, emotion and clinical symptoms), and analyzed the results of the two-dimensional and hierarchical model based on Chapman’s questionnaire. At the same time, we verified the applicability of our model to data by comparing different dimensional models.

## Methods

### Subjects

804 university students volunteered to participate in the study, and 56 questionnaires with missing values had been excluded. The final valid control sample was 748 people with mean age 21.27 ± 4.72 (350 women, 46.79%). 83 people with depression from three medical institutions participated in the study, and 3 questionnaires with missing values had been excluded. The final valid patients’ sample was 80 people aged 38.36 ± 16.42 (32 women, 40%). All the subjects were told that their answers are completely confidential. During the study, the patients’ emotional reaction and behavior were monitored by doctors and nurses, and the study was terminated immediately if there was any abnormal situation.

### Material

A demographic questionnaire collected data including age, gender, years of education, monthly family income, marital status and employment status.

The somatic anhedonia was measured by the PhAS (Physical Anhedonia Scale) which assessed the impairment of an individual’s ability to experience representative pleasurable physical stimuli, such as food, sex, and the higher scores indicated higher levels of somatic anhedonia. It includes 61 items, and its test-retest reliability is 0.87 and Cronbach’s α is 0.84 (Chapman, Chapman & Raulin, 1976).

The social anhedonia was measured by the SAS (Social Anhedonia Scale) which assessed the individual’s ability to experience non-physical stimuli, such as talking to others or communicating emotional expressions (Eckblad et al., 1982) And the higher scores indicated lower level of pleasure in social interaction. It has 40 items, and its test-retest reliability was 0.80 and Cronbach’s α was 0.82.

PANAS (Positive and Negative Affect Schedule) was used to assess individual’s positive and negative emotion (Watson, Clark & Tellegen, 1988). It has 20 words for positive emotion dimension and 20 words for negative emotion dimension. High positive emotion scores indicated an individual’s energetic, attentive and happy emotional state, while low scores indicate apathy. High scores of negative emotions indicated subjective feelings of confusion and distress, while low scores indicated calmness. In this study, the Cronbach’s α of positive emotion dimension was 0.86 and negative emotion dimension was 0.84.

Depression was measured by Beck Depression Inventory (BDI). BDI has 13 items and each item is assessed with a score of 0–3. In this study, the Cronbach’s α of BDI was 0.88.

### Statistical Analyses

**Figure 1:**
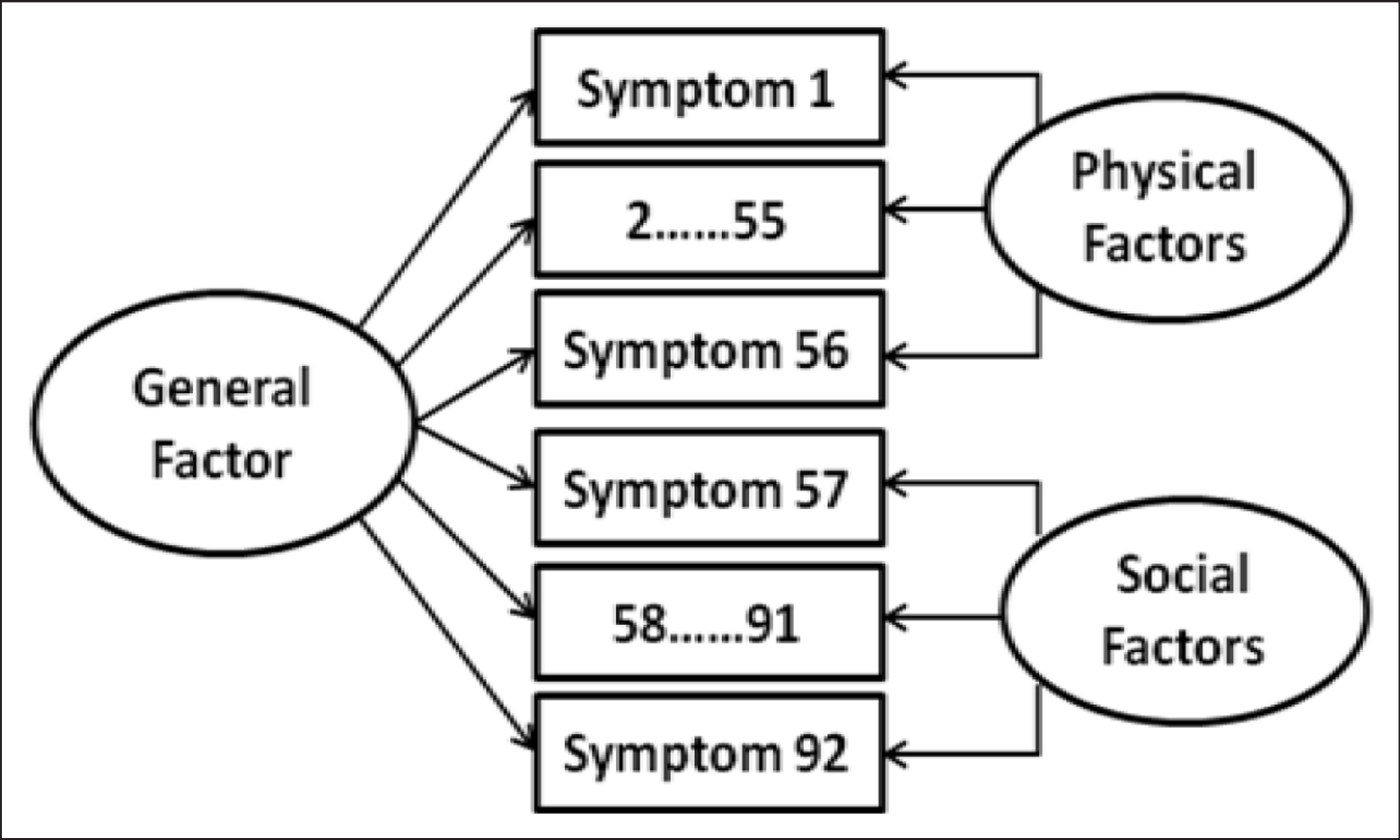
Bifactor structure model.

Classical measurement theory (CTT) and IRT were used to analyze the results of PhAS and SAS, and bifactor model was used to compare model fitting analysis, descriptive analysis was based on the original structure of PhA and SA. Then the changes in the original scale score, the general factor score and the special factor score were compared with a series of evaluation variables. The specific statistical analyses were based on our previous research (Xie et al., 2012). So, we can get the most accurate results of the severity of the scale. In order to prove the advantages of hierarchical bifactor model, we also analyzed several confirmatory factor analyses to compare the fitting degree of different models, including unidimensional (anhedonia), bidimensional (physical and social anhedonia), and bifactor model (general anhedonia, specific factors such as physical and social anhedonia). Bifactor model can be seen as a combination of single-dimension and two-dimension model, as shown in Figure 1.

Finally, the correlation between anhedonia factor and other psychological variables was tested, including positive and negative emotions, depressive symptoms, and the clinical evaluation of depression degree. All CTT analyses used SPSS23.0, and model fitting comparison was based on confirmatory factor analysis used Lisrel 8.50. Polybif software was used to calculate standardized scores of general factors and two special factors, as well as parameters of each item (Gibbons & Hedeker, 1992).

## Results

### Classical measurement theory analysis of the Chapman anhedonia scale

The score of PhAS of controls was significantly lower than in depression patients, and the score of SAS of controls was also significantly lower than in depression patients. See Table 1 for full results.

**Table 1:** Questionnaire scores as a function of group (mean and standard deviation).

Measurement	Group (x̄ ± s)		t	p

	Controls (n = 748)	Depression (n = 80)		

PhAS	17.97 (7.96)	26.60 (10.49)	8.91	<0.001
SAS	10.95 (5.77)	16.65 (8.20)	6.06	<0.001
PANAS				
PA	30.61 (6.75)	23.35 (6.92)	9.12	<0.001
NA	22.04 (6.17)	28.50 (7.39)	8.72	<0.001
BDI	6.67 (5.67)	14.20 (7.60)	10.88	<0.001

*Note*: PA is for positive affect, NA is for negative affect.

Next, we conducted correlation analysis among subscales, items and total scores. There was a significant positive correlation (r = 0.52, P < 0.01) between the two subscales of PhAS and SAS. The scores of items in PhAS and SAS were significantly correlated with the total scores of the other subscale, which were 94.64% and 91.67% respectively. The correlation coefficient (r_1_) between each item of PhAS and the total SAS score ranged from –0.12 to 0.33 (P < 0.01). And the correlation coefficient (r_1_) between each item of SAS and the total PhAS score ranged from 0.05 to 0.42 (P < 0.01). What’s more, the correlation between the two subscales in patients was higher, r_1_ ranged from –0.14 to 0.45 and r_2_ ranged from –0.05 to 0.51 (P < 0.01). These results implied that the items had multidimensional characteristics, so the multidimensional model should be suitable for the next analysis.

### IRT Bifactor analysis of the Chapman anhedonia questionnaire

**Figure 2:**
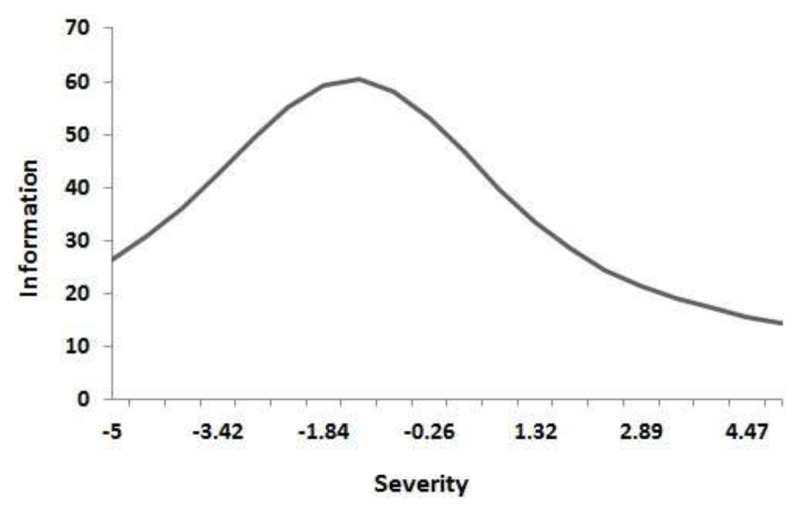
Bifactor analysis of information content of the test.

We conducted confirmatory two-factor model analysis on the original scale. As shown in Table 2, the load of 56 PhAS items and 36 SAS items was greater than 0.30. There were 28 items in PhAS (50.00%) and 23 items in SAS (63.89%) with the loading number of general factors greater than that of special factors, indicating that the severity of general factors (general anhedonia) evaluated in these items exceeded that of special anhedonia (namely, physical anhedonia or social anhedonia). The information content of the test (Figure 2) reflected the accuracy of the measurement of general factors by the anhedonia scale, and the information content was greater than 5 in a large range of subjects’ characteristics, which can better reflect the general hedonic impairment of the subjects.

**Table 2:** Factor loadings and severity of items in the somatic anhedonia scale.

	Item no. Abbreviated CRPA item	General loadings	Specific loadings	Symptom severity^a^

1	lovemaking usually be intensely pleasurable	**–0.308**	0.202	–0.472
2	enjoy the feel of silk et al.	–0.009	**0.467**	0.268
3	enjoy feeling the strength in muscles	0.091	**0.322**	0.160
4	dancing or the idea of it always dull	**0.498**	0.113	0.460
5	piano music dull and unexciting	**0.542**	0.094	0.756
6	the test of food has always been important	–0.011	**0.407**	0.539
7	have little fun from physical activities	**0.581**	0.022	0.747
8	seldom enjoy any kind of sexual experience	**0.459**	0.022	0.793
9	seldom want to sing along with a good song	**0.401**	0.069	0.638
10	always hated the feeling after vigorous activity	**0.463**	0.054	0.517
11	seldom matter the color painted on things	**0.339**	0.056	0.680
12	the sound of rustling leaves never pleased	**0.555**	0.152	0.766
13	prefer King down indoors to sunbathing	**0.520**	0.037	0.490
14	little things ever really enjoyed doing	**0.641**	–0.071	0.163
15	can’t know why people like music so much	**0.617**	0.040	0.764
16	flowers aren’t as beautiful as many people claim	**0.647**	0.114	0.591
17	always love having back massaged	0.075	**0.358**	0.044
18	never want to ride at an amusement park	**0.558**	0.114	0.798
19	always enjoy trying new food	0.297	0.515	0.732
20	warmth of an open fireplace cant sooth and calm	**0.472**	0.032	0.519
21	poets always exaggerate the good of nature	**0.382**	–0.078	–0.069
22	the urge to feel a statue	–0.061	**0.402**	0.081
23	a number of favorite food	0.215	**0.451**	0.577
24	why people enjoy looking at the stars	**0.637**	0.102	0.81
25	very little desire to take new kinds of food	**0.550**	0.229	0.853
26	never want to walk through a puddle barefoot	**0.344**	0.108	0.375
27	never care about the texture of food	**0.434**	0.029	0.535
28	the smell of fresh bread often make appetite	0.196	**0.494**	0.68
29	enjoy receiving a strong, warm handshake	**0.371**	**0.531**	1.111
30	friends touch often uncomfortable	**0.623**	0.064	0.926
31	standing tall and overlooking is very exciting	0.167	**0.508**	0.855
32	walks often is relaxing and enjoyable	**0.441**	**0.557**	1.051
33	the rain falling on the roof make snug and secure	0.221	**0.461**	0.388
34	like playing with and petting soft little pets	0.154	**0.394**	0.346
35	the sound of piano music has often thrilled	0.129	**0.428**	0.307
36	beautiful scenery has been a great delight	**0.477**	**0.615**	1.095
37	the first winter snowfall has often looked pretty	**0.449**	**0.463**	0.840
38	just feel the body move with the music sometimes	0.047	**0.352**	0.001
39	the impulse walk barefoot on a soft, thick carpet	0.102	**0.560**	0.570
40	a slow walk make relaxing after a busy day	**0.326**	**0.583**	0.809
41	exciting to look at the bright lights of a city	0.150	**0.497**	0.294
42	the beauty of sunsets is greatly overrated	**0.541**	–0.021	0.174
43	like someone care about reaches out to touch	0.299	**0.477**	0.950
44	usually find soft music boring	**0.594**	0.135	0.892
45	usually just to get bath or shower over with	**0.645**	0.065	0.84
46	the smell of dinner cooking hardly arouse appetite	**0.576**	0.125	0.907
47	often stop to smell flowers when pass by them	0.186	**0.434**	0.179
48	sex is the most intensely enjoyable thing in life	**–0.357**	0.256	–0.691
49	flying a kite is silly	**0.684**	0.119	1.039
50	never care to sunbathe	**0.618**	0.098	0.783
51	the sounds of a parade never excite	**0.607**	0.095	0.742
52	massage feel good when muscles tire or sore	0.254	**0.449**	0.847
53	singing often make happier when feel a little sad	**0.403**	**0.512**	0.785
54	A good soap lather make sooth and refresh	0.129	**0.517**	0.234
55	A brisk walk make feel good all over	**0.487**	**0.537**	1.059
56	fascinate with dancing of flames in a fireplace	–0.150	**0.528**	0.214
57	close friend is not as important as people say	**0.600**	0.130	0.888
58	having close friends is not very important	**0.656**	0.27	1.236
59	prefer watching tv to going with other people	**0.685**	0.12	0.754
60	ride car with someone is much more enjoyable	**0.354**	**0.555**	0.834
61	like to make long distance phone calls	**0.351**	**0.489**	0.521
62	playing with children is a real chore	**0.542**	0.041	0.553
63	always enjoy looking at friends’ photographs	0.288	**0.555**	0.808
64	have more fun doing things with other people	**0.398**	**0.541**	0.869
65	deeply attach to people often along with	–0.276	**0.444**	0.419
66	not shy but just want to be left alone	**0.377**	–0.076	–0.356
67	feel good too when close friends things go good	**0.416**	**0.566**	1.107
68	feel down too when close friends are depressed	0.086	0.377	0. 616
69	emotional responses very different from others	**0.516**	–0.015	0. 223
70	resent being disturbed when be alone	**0.659**	0.031	0.715
71	feel good being with friends	**0.499**	**0.688**	1. 229
72	like to talk to other people about bothering things	**0.443**	0.279	0.545
73	prefer hobbies and leisure activities alone	**0.472**	0.001	0. 295
74	it’s fun to sing with other people	**0.532**	**0.466**	0.869
75	sense of security from friends’ care	**0.336**	**0.607**	1.054
76	need to make new friends when move to new place	**0.339**	**0.484**	0.95
77	don’t really feel affection for certain people	**0.340**	–0.134	–0.07
78	people often expect too much time to talk with them	**0.449**	–0.117	0.220
79	feel good as learn more about friends’ emotion life	**0.338**	**0.523**	0.813
80	listen with interest and attention of others’ problems	**0.473**	**0.507**	1.107
81	never had really close friends in high school	**0.633**	0.192	1.075
82	content to just sit alone, thinking and day dream	**0.508**	–0.155	–0.130
83	too independent to involved with other people	**0.740**	0.083	0.679
84	a long, personal discussion is most boring	**0.504**	–0.055	0.315
85	sad to see high school friends go separate ways	0.108	**0.444**	0.333
86	hard to resist talking even when other things to do	–0.009	**0.489**	0.304
87	no worth to make new friends	**0.701**	0.185	1.172
88	people usually give up getting to know me better	**0.577**	0.039	0.359
89	happy living alone in the woods or mountains	**0.474**	0.051	0.453
90	rather to be with others than be alone	**0.406**	**0.393**	0.435
91	don’t really feel very close to friends	**0.620**	0.178	0.645
92	prefer the company of pets to the company of people	**0.636**	0.073	0.891

*Note*: All samples (N = 828) are used for parameter estimation. ^a^ Standardized entry difficulty and critical parameters.

We furthermore calculated the correlations between clinical indicators and anhedonia. After the consideration of general factors, the clinical indicators of the clinical patient group and biPhA and biSA were no longer correlated, as shown in Table 3.

**Table 3:** Correlations between the different questionnaire scores as a function of group.

	Controls (n = 748)				Depression (n = 80)			

	PhAS	biPhAS	SAS	biSAS	PhAS	biPhA	SAS	biSAS

PANAS								
PA	–0.34**	–0.29**	–0.29**	–0.08*	–0.45**	–0.22	–0.28	–0.06
NA	0.26**	0.04	0.29**	0.04	0.37*	0.04	0.50**	0.04
BDI	0.36**	0.09*	0.43**	0.08*	0.45**	0.17	0.28	0.17

*Note*: PhA is for physical anhedonia, SA is for social anhedonia, PA is for positive affect, NA is for negative affect. biPA and biSA were obtained by bifactor model analysis after considering general factors. * P < 0.05, ** P < 0.01.

Furthermore, all clinical indicators significantly correlated with the general factor, as shown in Table 4.

**Table 4:** Descriptive statistics and correlations for the general factor (x̄ ± s, r).

Clinical indicators	General factor	

	Controls (n = 748)	Depression (n = 80)

	–0.11 ± 0.78	0.60 ± 0.83
PA	–0.33**	–0.37*
NA	0.35**	0.62*
BDI	0.47**	0.48**

*Note*: PA is for positive affect, NA is for negative affect. * P < 0.05, ** P < 0.01.

### Model fitting analysis

The fitting indexes of different models were compared to verify the superiority of the bifactor model, as shown in Table 5. The results showed that the bifactor model was better than the single-dimension and two-dimension models. According to the original structure of the scale, the bifactor model integrated the single-dimension model and the two-dimensional model. The results confirmed that the bifactor model provided a more consistent model with the observed values than the one-dimensional factor model.

**Table 5:** Model fitting analysis.

Model	CFI	GFI	RMSEA	χ^2^	df	Δχ^2^	Δdf

Unidimensional model	0.71	0.79	0.04	14693.70	4949	7083.26**	95
Bidimensional model	0.70	0.79	0.04	14716.67	4957	7106.23**	103
Bifactor model	0.90	0.90	0.02	7610.44	4854	–	–

*Note*: * P < 0.05; ** P < 0.01. Δχ^2^ and Δdf represent fitting comparison in the one-dimensional model, two-dimensional model and bifactor model.

## Discussion

In this study, the theoretical composition of Chapman’s anhedonia questionnaire was quantitatively analyzed with the bifactor model. Different from the traditional physical anhedonia and social anhedonia approach, this study showed that the anhedonia components in both dimensions had multidimensional characteristics, including general factors and two special factors (biPhA and biSA). Compared with the traditional CTT analysis method, IRT analysis was also sensitive to the overall differences between non-clinical samples and depressed patients, showing significant differences in general factors and two special factors. In addition, IRT was more accurate in reflecting the anhedonia characteristics of depression patients, that is, anhedonia and negative affect of depression patients coexist and interact with each other.

Secondly, the specific performance of general factors in depression patients can distinguish the severity and characteristics of them. Anhedonia of depressed patients was significantly correlated with emotional state and negative symptom level, which was consistent with the conclusion of previous studies. Cognitive Theory of Depression proposed that there was a cognitive bias in patients with severe depression, and the negative cognitive bias was associated with the severity of depression (Haaga & Beck, 1995; Mckendree-Smith & Scogin, 2000). Results of bifactor model showed that the general factor of depression patients was significantly related with positive affect, negative affect and BDI, which indicates that the level of pleasure lack of depression patients marks the clinical severity of disease, and this may be on behalf of a special kind of mental state of the experience of the pathology. This vulnerability trait was also reflected in controls, and there was a significant positive correlation between general factors and subjective depression assessment (BDI) in non-clinical samples, which can play a role in the assessment of prodromal or early symptoms of depressive disorder, which was also consistent with the view of depression vulnerability model (Gwenolé Loas, 1996). This suggests that in the treatment of patients with depression, we should not only focus on the improvement of negative emotions, but also help them to strengthen the perception of positive emotions, especially the improvement of social pleasure sensitivity, which will have a positive impact on the treatment and recovery of patients with depression.

Anhedonia is related to a series of dysfunctions of individual functional systems, including expectation, emotion management, memory, motivation, learning and social function. Anhedonia may be comprehensive or specific, including sensory and motor impairments, and may occur outside the field of psychopathology or disease. Bifactor model analysis provides a better understanding of anhedonia in the field of depression and it may indicate the most effective way to treat anhedonia in the future.
